# Reduced lethality in mice receiving a combined dose of cyclophosphamide and busulphan.

**DOI:** 10.1038/bjc.1975.149

**Published:** 1975-08

**Authors:** J. L. Millar, B. N. Hudspith, N. M. Blackett

## Abstract

Animals treated with a sufficiently high dose of busulphan die about 14 days later from bone marrow failure. A single, appropriately timed injection of cyclophosphamide can save these mice. The nature of this protection is shown to be the cyclophosphamide induced elaboration of a humoral factor which stimulates haemopoietic recovery.


Br. J. Cancer (1975) 32, 193

### REDUCED LETHALITY IN MICE RECEIVING A COMBINED DOSE OF CYCLOPHOSPHAMIDE AND BUSULPHAN

J. L. MILLAR, B. N. HUDSPITH AND N. M. BLACKETT

*From the Biophysics Division, Institute of Cancer Research, Sutton, Surrey SM2 5PX*

Received 31 January 1975. Accepted 2 May 1975

**Summary.—Animals treated with a sufficiently high dose of busulphan die about 14 days later from bone marrow failure. A single, appropriately timed injection of cyclophosphamide can save these mice. The nature of this protection is shown to be the cyclophosphamide induced elaboration of a humoral factor which stimulates haemopoietic recovery.**

THE USE of cytotoxic agents in cancer chemotherapy is often limited by the action of these agents on the normal, homoeostatic, proliferating cells of the body, particularly those of the bone marrow. Any substance which enhances haemopoietic recovery, particularly during bone marrow depression induced by cytotoxic agents, is of importance.

Cyclophosphamide, in addition to having a cytotoxic effect on the proliferating haemopoietic cells of the bone marrow, appears also to enhance subsequent recovery of these cells (Gregory *et al.*, 1971; Fried *et al.*, 1973). Evidence that a long-range diffusible substance is involved in this stimulation was presented by Stohlman *et al.* (1973). These authors observed enhanced growth of bone marrow in diffusion chambers implanted in cyclophosphamide treated hosts.

The timing of the administration of cyclophosphamide is important in relation to the transplantation of the bone marrow (Gregory *et al.*, 1971; Fried *et al.*, 1973) the cyclophosphamide being given between 1 and 4 days before the transplantation for optimum stimulation. Gregory *et al.* (1971) have suggested that the cyclophosphamide may cause the elaboration of a substance as a result of cell damage which stimulates haemopoietic stem cell recovery. Dons *et al.* (1974) have shown that factor(s) present in spleen extract and fetuin, a foetal protein, cause regeneration of the haemopoietic stem cells in irradiated mice.

In this communication direct evidence is presented that haemopoiesis is enhanced by a humoral factor elaborated after cyclophosphamide administration. It is demonstrated that this serum factor is transferable and can prevent the death of otherwise lethally treated animals.

### MATERIALS AND METHODS

*Animals.*—Male or female, 9–10 week old, CBA mice were used in all experiments. The female mice, weighing between 18 and 23 g acted as recipients of bone marrow in spleen colony experiments. The male mice, weighing between 22 and 27 g, were used in all other experiments. Both male and female mice were used to obtain serum.

*Drugs.*—Cyclophosphamide (Endoxana, Ward Blenkinsop & Co.) was dissolved in water for injection, whereas the busulphan (Burroughs Wellcome & Co.) was first dissolved in dimethyl sulphoxide to form a 0·05% (w/v) solution, which was then emulsified in arachis oil in the ratio of 9 parts arachis oil to 1 part solution. Both drugs were injected intraperitoneally.

*CFU assay.*—The CFU content of femurs was assayed using the method of Till and McCulloch (1961). Recipient mice were given 850 rad $^{60}$Co $\gamma$ irradiation 4 h before bone marrow transplantation. Groups varied in size from 8 to 14 mice.

*Granulocyte count.*—The blood leucocyte concentration was measured using a model F

Coulter counter and differentials performed on ethanol fixed, Giemsa stained blood films made at the time of sampling. A hundred cells per slide were analysed.

*Serum.*—Mice were given 200 mg/kg cyclophosphamide and 2 days later bled from the axilla. The blood was allowed to clot overnight at 4 °C. After centrifugation the serum was removed, passed through a 0·22 $\mu$m Millipore filter and dialysed in 8/32 Visking tubing against distilled water for 3 days at 4 °C. The water, which contained 250,000 u of both penicillin and streptomycin per litre, was changed twice during the dialysis. The product was centrifuged to remove the precipitate, which was resuspended in isotonic saline to its original concentration. Test sera were injected intraperitoneally in 0·5 ml aliquots daily for three days following the busulphan.

### RESULTS

### *Effect of the combination of cyclophosphamide and busulphan on the primary animal*

*Animal survival.*—Cyclophosphamide (200 mg/kg) was given to animals lethally treated with busulphan (35 mg/kg or 45 mg/kg) at different times before or after the busulphan (Table Ia). These doses of busulphan result in the death of at least 90% of the treated animals in 30 days but the dose of cyclophosphamide used has never produced any deaths over the 30-day period investigated. The survival of animals that received the lethal dose of busulphan as well as the cyclophosphamide improved dramatically when the cyclophosphamide was injected one or 2 days before the busulphan. At other times the survival was less, but generally better than that of animals receiving no cyclophosphamide. Table Ib indicates that the spleen does not play a major role in this effect. Using the optimum timing and order of administration of the drugs, it was shown that the considerable sparing effect seen in Table Ia could be obtained in animals recently splenectomized or splenectomized for some time compared with similar animals receiving busulphan alone.

*Effect on blood granulocyte concentration.*—The effect of cyclophosphamide (200 mg/kg), busulphan (30 mg/kg) and a combination of the two drugs on the time course of blood granulocyte concentration is shown in the Figure. The combination, cyclophosphamide given 48 h before busulphan, is that which gives the best survival as judged by Table Ia.

A reduced dose of busulphan (30 mg/kg) was used to allow 21-day survival in groups of animals given busulphan alone. An animal receiving this drug alone initially maintains a high blood granulocyte concentration (Figure); the granulocyte concentration then falls, there is an abortive recovery Day 7 to 9 and finally it reaches its lowest value after 14 days when

TABLE Ia.—*Effect of Pre- or Post Treatment with Cyclophosphamide (200 mg/kg) on the 30-day Survival of Animals Lethally Treated with Busulphan (35 mg/kg or 45 mg/kg)*

| Time of cyclophosphamide injection in relation to busulphan | 30-day survival after 35 mg/kg Bu | | 30-day survival after 45 mg/kg Bu | | Average day of death of non-survivors ± S.E. |
|---|---|---|---|---|---|
| 4 days before | 1/14 | 7% | 0/15 | 0% | 11·9±0·4 |
| 3 days before | | | 0/14 | 0% | 12·9±1·1 |
| 2 days before | 13/14 | 93% | 6/14 | 43% | 21·9±1·5 |
| 1 day before | 12/14 | 86% | 9/14 | 64% | 17·7±2·2 |
| same day | 10/14 | 71% | 5/14 | 36% | 11·5±2·4 |
| 1 day after | 10/14 | 71% | 4/14 | 29% | 13·6±1·1 |
| 2 days after | | | 2/14 | 14% | 12·8±1·0 |
| 3 days after | 5/14 | 36% | 1/14 | 7% | 14·2±1·6 |
| 4 days after | | | 2/14 | 14% | 14·2±0·6 |
| Busulphan only controls | 0/14 | 0% | 0/14 | 0% | 15·8±0·9 |
| Animals receiving cyclophosphamide only | 14/14 | 100% | 14/14 | 100% | |

TABLE Ib.—*Effect of Splenectomy on the Enhanced Survival of Animals Given Cy (200 mg/kg) One Day Before a Lethal Dose of Bu (45 mg/kg)*

| Animals splenectomized | 30-day survival after Cy (200 mg/kg) 1day before Bu (45 mg/kg) | | 30-day survival after Bu alone (45 mg/kg) | |
|---|---|---|---|---|
| 5 months before treatment | 3/5 | 60% | 0/5 | 0% |
| 2 weeks before treatment | 5/9 | 56% | 1/8 | 13% |

FIGURE.—Recovery of peripheral granulocytes after treatment with 200 mg/kg cyclophosphamide (Cy), 30 mg/kg busulphan (Bu) or both. Cy48Bu indicates that the cyclophosphamide was injected 48 h before the busulphan. Note that the time scale refers to the busulphan injection.

the animal usually dies. In contrast, an animal given cyclophosphamide alone experiences a rapid initial fall in blood granulocyte level followed by a very rapid recovery from Day 4 to Day 7, after which there is a period of neutrophilia lasting 6–7 days. The blood granulocyte level in an animal that received the drug combination (cyclophosphamide 2 days before busulphan) behaves very similarly to the cyclophosphamide alone situation, suggesting that cyclophosphamide pretreatment was rendering the busulphan ineffective. For this reason, in all the serum experiments to be described next busulphan was administered 24 h before the serum.

### *Experiments involving serum from cyclophosphamide treated animals*

*Effect on survival of busulphan treated mice.*—Serum collected from mice 2 days after treatment with cyclophosphamide (200 mg/kg) was injected into syngeneic mice lethally treated with busulphan (40 mg/kg). Table II shows that dialysed serum injected i.p. on 3 consecutive days following the busulphan administration markedly increased the 30-day survival

TABLE II.—*Effect of Serum from Cyclophosphamide Treated Animals on the 30-day Survival of Animals Lethally Treated with Busulphan* (40 *mg/kg*)

| Animals treated with 40 mg/kg busulphan and: | 30-day survival | | Average day of death of non-survivors ± S.E. | Average granulocyte count per mm³ 10 days after busulphan ± S.E. |
|---|---|---|---|---|
| Normal serum | 0/13 | 0% | 14·8±0·7 | 609±137 |
| Normal serum after dialysis | 1/25 | 4% | 15·1±0·7 | 152±26 |
| Precipitate from dialysed normal serum | 1/23 | 4% | 14·6±0·7 | 405±144 |
| Serum from animals pretreated with cyclophosphamide | 5/14 | 36% | 14·7±1·0 | 1018±292 |
| Serum from animals pretreated with cyclophosphamide after dialysis | 11/24 | 46% | 18·4±0·6 | 2103±430 |
| Precipitate from dialysed serum of animals pretreated with cyclophosphamide | 3/25 | 12% | 16·4±0·6 | 1160±272 |
| No serum | 1/24 | 4% | 16·0±0·9 | 1656±349 |

TABLE III.—*Effect of Serum from Cyclophosphamide Treated Animals on the Femoral CFU Content of Animals Lethally Treated with Busulphan* (40 *mg/kg*), *Expressed as a Percentage of Control*

| Experiment | 40 mg/kg busulphan ± normal dialysed serum | 40 mg/kg busulphan + dialysed serum from cyclophosphamide treated animals | 40 mg/kg busulphan alone |
|---|---|---|---|
| *Exp. A* | | | |
| Normal animals 7 days recovery | 0·51±0·06% | 0·15±0·05% | 1·4±0·14% |
| *Exp. B* | | | |
| Normal animals | | | |
| 4-day recovery | 4·3±0·9% | 5·3±0·6% | 6·5±0·6% |
| 7-day recovery | 8·5±0·6% | 6·4±0·5% | 9·0±0·8% |
| *Exp. C* | | | |
| Splenectomized animals | | | |
| 7-day recovery | 0·30±0·05% | 0·15±0·04% | 0·09±0·03% |
| 14-day recovery | 0·12±0·04% | 0·52±0·09% | 0·39±0·09% |
| *Exp. D* | | | |
| Normal animals | | | |
| 11-day recovery | 0·19±0·05% | 0·14±0·05% | 0·33±0·07% |
| 14-day recovery | 0·27±0·06% | 0·32±0·08% | 0·48±0·09% |
| Splenectomized animals | | | |
| 11-day recovery | 0·35±0·07% | 0·32±0·08% | 0·46±0·10% |
| 14-day recovery | 0·52±0·11% | 0·41±0·09% | 0·65±0·12% |

compared with dialysed normal serum. The peripheral blood granulocyte count 10 days after the busulphan demonstrates that the animals which received serum from cyclophosphamide treated animals, particularly those that received the dialysed serum, had considerably higher granulocyte counts than those which received normal serum, although they were not much higher than the count in animals receiving no serum at all.

*Effect on CFU*.—Table III shows the CFU concentration in femurs of mice treated with a lethal dose of busulphan and with dialysed serum from normal or cyclophosphamide pretreated animals. The experiments, performed in both normal and splenectomized animals various days after treatment with busulphan and serum, show that the serum had no stimulating effect on CFU recovery, whether it came from normal animals or from animals given cyclophosphamide. Indeed, in many instances animals given no serum at all have higher CFU levels per femur than those given serum.

### DISCUSSION

The improved survival of mice treated with busulphan and cyclophosphamide is greatest when the cyclophosphamide is given 1–2 days before the busulphan. However, there is still improved survival when cyclophosphamide is given after the busulphan, indicating that the improved survival is not simply a result of the cyclophosphamide interfering with the action of busulphan.

Cyclophosphamide has been shown to enhance the regeneration of transplanted CFU (Fried and Johnson, 1968; Gregory *et al.*, 1971) although the mechanism of this effect remains unclear. Fried *et al.* (1973) failed to demonstrate the presence of a humoral factor in plasma from animals treated with cyclophosphamide which could stimulate CFU regeneration in irradiated animals. They concluded that cyclophosphamide may have its effect at a local level by destroying many of the bone marrow cells and thus removing cell–cell contact inhibition.

We, too, have failed to demonstrate a serum factor capable of restoring CFU number even though the serum does contain a factor which will increase blood granulocyte concentration and improve survival of lethally treated mice. However, other workers have reported that the recovery of stem cells (CFU) has been influenced by the administration of various substances. An alpha macroglobulin prepared from mouse, or rat serum (Nettesheim, Hanna and Fisher, 1968) and cell-free spleen extracts are reported to enhance CFU regeneration in mice given 200 rad (Knospe *et al.*, 1970). Most recently, fetuin, an alpha macroglobulin extracted from foetal calf serum, has been shown to enhance CFU regeneration in sub-lethally irradiated mice (Dons *et al.*, 1974).

It seems likely therefore that humoral stimulators of granulopoiesis exist which restore granulopoiesis after damage by irradiation or cytotoxic drugs and that this is not confined to restoration of the stem cell pool but also enhancement of differentiation along the granulocytic pathway. An animal's survival does not depend on stem cells alone but on the capacity of these cells to form functional progeny and the degree of stimulus to do so. This is borne out by the disparity between CFU content of the femur and actual survival of the animal after various cytotoxic treatments seen by other workers (Hanks and Ainsworth, 1964; Smith *et al.*, 1966; Yuhas and Storer, 1969; Dunn and Elson, 1970; Dunjic and Cuvelier, 1973), and this emphasizes the limitation of predicting haemopoietic recovery in terms of CFU measurement only.

An important question arises as to the relationship between the factor reported here and CSF, the factor necessary for the *in vitro* growth of granulocytic and monocytic colonies. It has been shown that there is a rapid increase in CSF in the serum of animals treated with bacterial endotoxin (Quesenberry *et al.*, 1972). In our system the action of cyclophosphamide on the cells of the intestinal epithelium may have led to a cellular breakdown and bacterial invasion from the gut flora. The subsequent bacteriaemia could have led to high levels of CSF at the time when serum was collected for analysis.

The presence of high levels of CSF in the serum 2 days after cyclophosphamide is unlikely, however, as it has not been detected under these conditions by Shadduck and Nagabhushanam (1971) or by us (unpublished observations) using the *in vitro* colony forming assay (Pluznik and Sachs, 1965; Bradley and Metcalf, 1966). Nevertheless, work is in progress to assess the effects of bacterial endotoxin and endotoxin treated mouse serum in sparing animals from busulphan induced bone marrow failure. The work started by Smith, Alderman and Gillespie (1957) and Hanks and Ainsworth (1964) has made this an important consideration.

### REFERENCES

Bradley, T. R. & Metcalf, D. (1966) The Growth of Mouse Bone Marrow Cells *in vitro*. *Aust. J. exp. Biol. med. Sci.*, **44**, 287.

Dons, R., Knospe, W. H., Trobaugh, F. E. Jr & Fried, W. (1974) The Effect of Fetuin and of Spleen Extracts on Haematopoietic Precursors and on Differentiated Hematopoietic Cells in Irradiated Mice. *Cell tissue Kinet.*, **7**, 371.

DUNJIC, A. & CUVELIER, A-M. (1973) Survival of Rat Bone Marrow Cells after Treatment with Myleran and Endoxan. *Expl Hemat.*, **1**, 11.

DUNN, C. D. R. & ELSON, L. A. (1970) The Effect of a Homologous Series of Dimethane-sulphonoxy-alkanes on Haemopoietic Colony Forming Units in the Rat. *Chem. biol. Interact.*, **2**, 273.

FRIED, W. & JOHNSON, C. (1968) The Effect of Cyclophosphamide on Hematopoietic Stem Cells. *Radiat. Res.*, **36**, 521.

FRIED, W., HUSSEINI, S., GREGORY, S., KNOSPE, W. H. & TROBAUGH, F. E. JR (1973) Effect of Cyclophosphamide on the Haematopoietic Microenvironmental Factors which Influence Haemopoietic Stem Cell Proliferation. *Cell tissue Kinet.*, **6**, 155.

GREGORY, S. A., FRIED, W., KNOSPE, W. H. & TROBAUGH, F. E. JR (1971) Accelerated Regeneration of Transplanted Hematopoietic Stem Cells in Irradiated Mice Pretreated with Cyclophosphamide. *Blood*, **37**, 196.

HANKS, G. E. & AINSWORTH, E. J. (1964) Endotoxin Protection and Colony-forming Units. *Radiat. Res.*, **32**, 367.

KNOSPE, W. H., FRIED, W., GREGORY, S. A., SASSETTI, R. J. & TROBAUGH, F. E. JR (1970) Effect of a Noncellular Spleen-derived Factor on Recovery of Haemopoietic Stem Cell from Irradiation. *J. Lab. clin. Med.*, **76**, 585.

NETTESHEIM, P., HANNA, M. G. JR & FISHER, P. (1968) Further Studies on the Effect of Serum Alpha Macroglobulin on Regeneration of Haemopoietic Tissue after X-irradiation. *Radiat. Res.*, **35**, 378.

PLUZNIK, D. H. & SACHS, L. (1965) The Cloning of Natural " Mast " Cells in Tissue Culture. *J. cell Comp. Physiol.*, **66**, 319.

QUESENBERRY, P., MORLEY, A., STOHLMAN, F. JR, RICKARD, K., HOWARD, D. & SMITH, M. (1972) The Effect of Endotoxin upon Granulopoiesis. I. Colony Stimulating Factor. *New Engl. J. Med.*, **286**, 227.

SHADDUCK, R. K. & NAGABHUSHANAM, N. G. (1971) Granulocyte Colony Stimulating Factor. I. Response to Acute Granulocytopenia. *Blood*, **38**, 559.

SMITH, W. W., ALDERMAN, I. M. & GILLESPIE, R. E. (1957) Increased Survival in IrradiatedAnimals Treated with Bacterial Endotoxin. *Am. J. Physiol.*, **191**, 124.

SMITH, W. W., BRECHER, G., BUDD, R. A. & FRED, A. (1966) Effects of Bacteiial Endotoxin on the Occurrence of Spleen Colonies in Irradiated Mice. *Radiat. Res.*, **27**, 369.

STOHLMAN, F. JR, QUESENBERRY, P., NISKANIN, E., MORLEY, A., TYLER, W., RICHARD, K., SYMANN, M., MONETTE, F. & HOWARD, D. (1973) Control of Granulopoiesis. In *Haemopoietic Stem Cells*. Eds. G. E. W. Wolstenholm and M. O'Connor. Amsterdam: CIBA Foundation Symposium, (New Series) 13.

TILL, J. E. & MCCULLOCH, E. A. (1961) A Direct Measurement of the Radiation Sensitivity of Normal Mouse Bone Marrow Cells. *Radiat. Res.*, **14**, 213.

YUHAS, J. M. & STORER, J. B. (1969) On Mouse Strain Differences in Radiation Resistance: Haemopoietic Death and Endogenous Colony-forming Units. *Radiat. Res.*, **39**, 608.


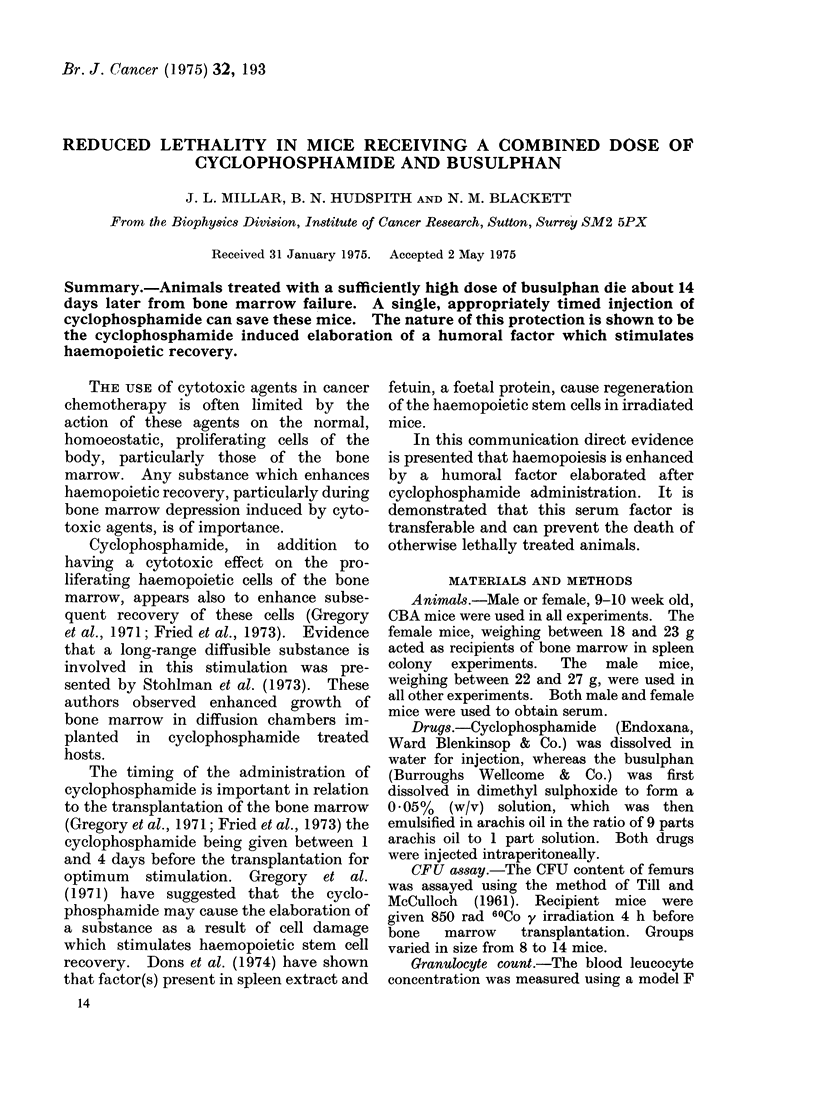

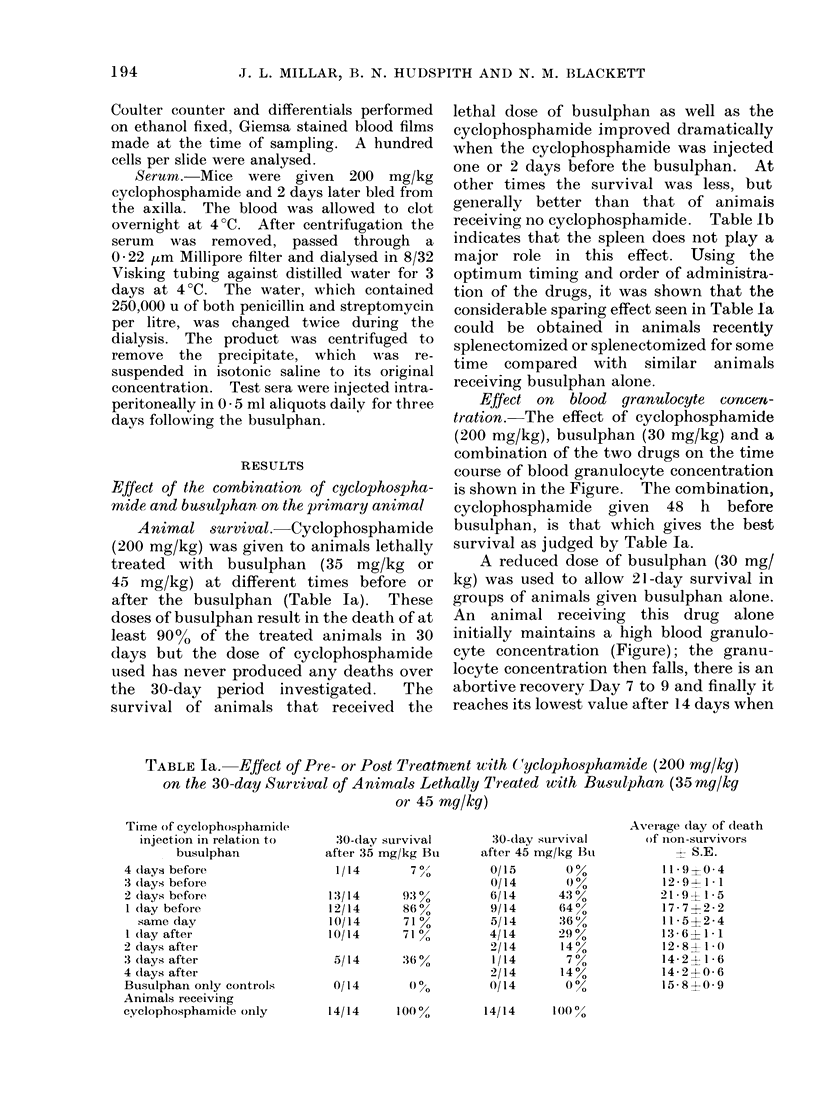

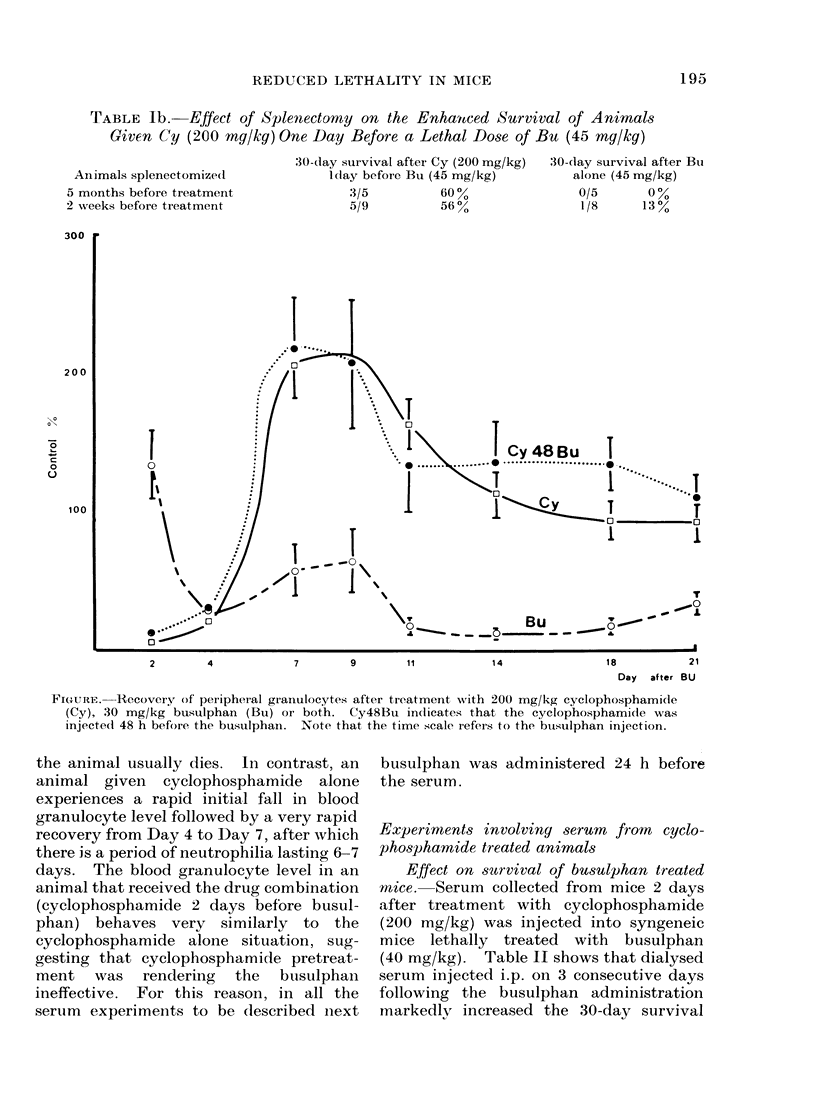

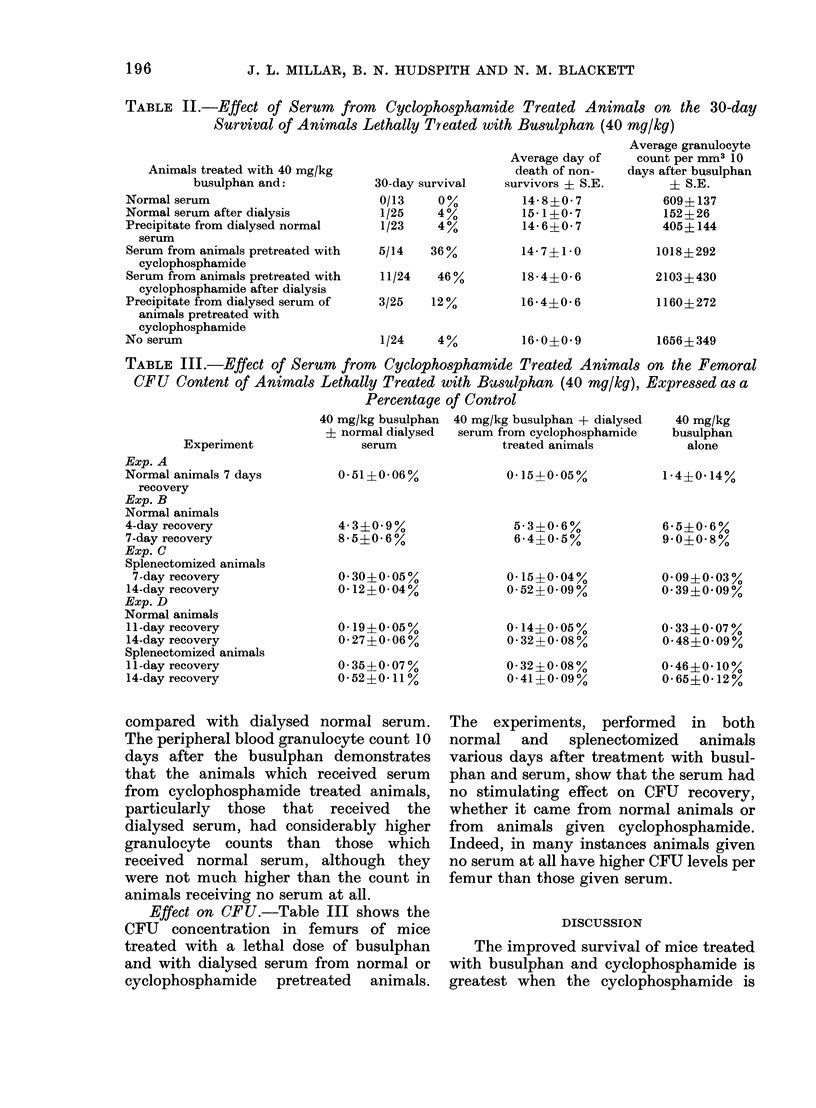

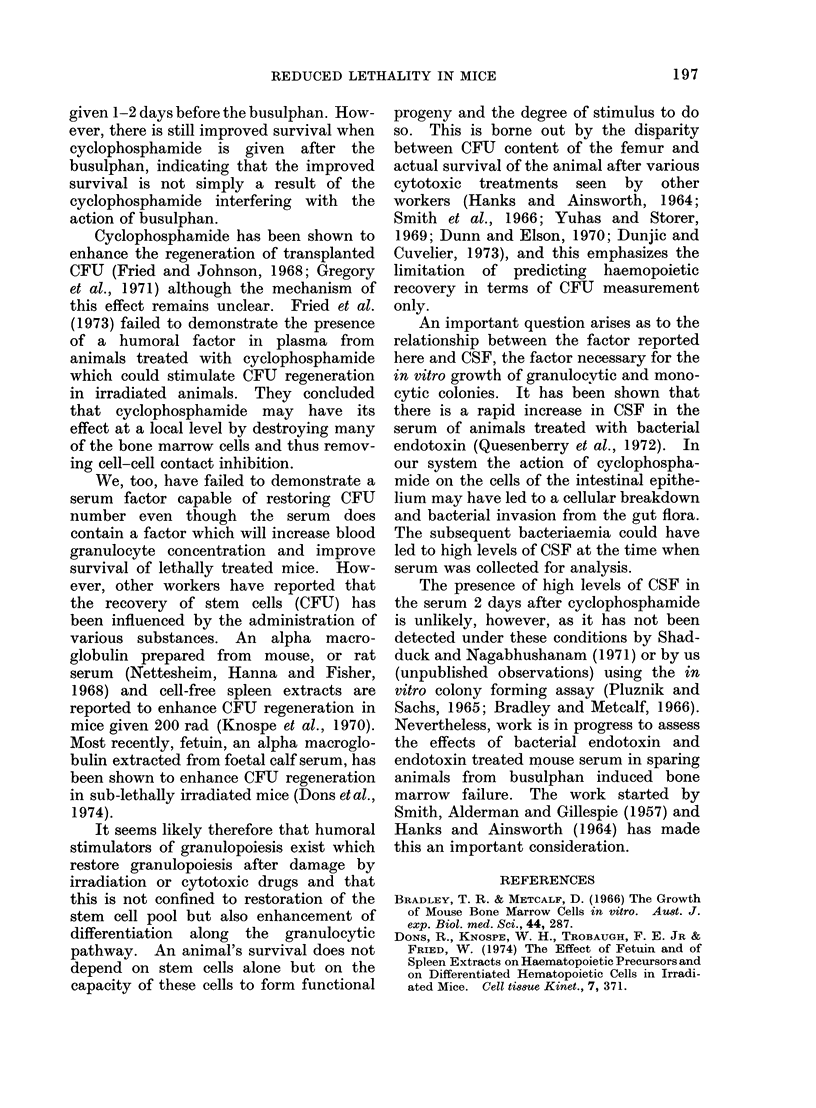

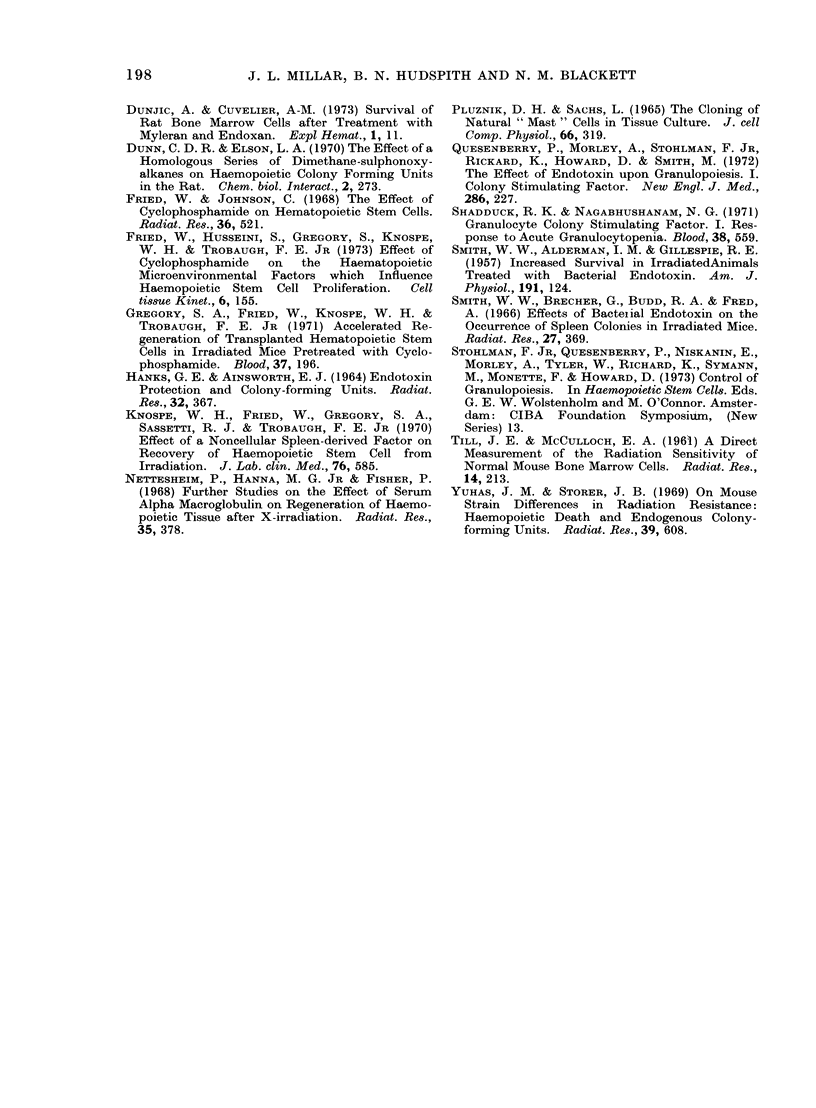


## References

[OCR_00657] Bradley T. R., Metcalf D. (1966). The growth of mouse bone marrow cells in vitro.. Aust J Exp Biol Med Sci.

[OCR_00662] Dons R., Knospe W. H., Trobaugh F. E., Fried W. (1974). The effect of fetuin and of spleen extract on hematopoietic precursors and on differentiated hematopoietic cells in irradiated mice.. Cell Tissue Kinet.

[OCR_00671] Dunjic A., Cuvelier A. M. (1973). Survival of rat bone marrow cells after treatment with myleran and endoxan.. Exp Hematol.

[OCR_00676] Dunn C. D., Elson L. A. (1970). The effect of a homologous series of dimethane-sulphonoxy-alkanes on haemopoietic colony forming units in the rat.. Chem Biol Interact.

[OCR_00687] Fried W., Husseini S., Gregory S., Knospe W. H., Trobaugh F. E. (1973). Effect of cyclophosphamide on the hematopoietic microenvironmental factors which influence hematopoietic stem cell proliferation.. Cell Tissue Kinet.

[OCR_00682] Fried W., Johnson C. (1968). The effect of cyclophosphamide on hematopoietic stem cells.. Radiat Res.

[OCR_00695] Gregory S. A., Fried W., Knospe W. H., Trobaugh F. E. (1971). Accelerated regeneration of transplanted hematopoietic stem cells in irradiated mice pretreated with cyclophosphamide.. Blood.

[OCR_00702] Hanks G. E., Ainsworth E. J. (1967). Endotoxin protection and colony-forming units.. Radiat Res.

[OCR_00721] Pluznik D. H., Sachs L. (1965). The cloning of normal "mast" cells in tissue culture.. J Cell Physiol.

[OCR_00726] Quesenberry P., Morley A., Stohlman F., Rickard K., Howard D., Smith M. (1972). Effect of endotoxin on granulopoiesis and colony-stimulating factor.. N Engl J Med.

[OCR_00737] SMITH W. W., ALDERMAN I. M., GILLESPIE R. E. (1957). Increased survival in irradiated animals treated with bacterial endotoxins.. Am J Physiol.

[OCR_00733] Shadduck R. K., Nagabhushanam N. G. (1971). Granulocyte colony stimulating factor. I. Response to acute granulocytopenia.. Blood.

[OCR_00743] Smith W. W., Brecher G., Budd R. A., Fred S. (1966). Effects of bacterial endotoxin on the occurrence of spleen colonies in irradiated mice.. Radiat Res.

[OCR_00764] Yuhas J. M., Storer J. B. (1969). On mouse strain differences in radiation resistance: hematopoietic death and the endogenous colony-forming unit.. Radiat Res.

